# A case of impacted foreign body in the rectum that was extracted using size 24 Foley catheter

**DOI:** 10.1016/j.ijscr.2019.11.035

**Published:** 2019-12-04

**Authors:** Imad A. Bakheit, Gazali A.B. Elhasan, Mugahid A. Salih

**Affiliations:** a Faculty of Medicine, University of Khartoum, Sudan; b General Surgery Resident, Sudan

**Keywords:** Rectum, Foreign body, Foley catheter

## Abstract

**Introduction:**

Retained foreign bodies in the rectum represent a common challenge to clinicians. They have different modes and reasons for insertion. Many case series and case reports described several methods for removal of these foreign bodies.

**Case report:**

This is a case of a 61 –year – old male who presented to the surgical outpatient with anal pain and bleeding. On digital rectal examination the bottom of a foreign body was palpable. Under general anesthesia a trial of extraction using forceps failed, thus, a size 24 Foley catheter was used to remove the foreign body. It was a big long perfume bottle. The patient had an uneventful recovery period and he was followed for two weeks. No complications developed.

**Conclusion:**

Foreign bodies in the rectum are not uncommon. Many methods can be used for their removal and Foley catheter extraction is one of them.

## Highlights

• Foreign bodies in the rectum are not uncommon.
• Retained foreign bodies in the rectum are a common challenge to clinicians.
• Many noninvasive and invasive surgical methods can be used for their removal.
• The use of a size 24 Foley catheter can be a safe method for removal of these kind of foreign bodies.

## 1 Introduction and literature review

This work has been reported in line with the SCARE criteria [1].

One of the serious challenges that faces clinicians is impacted foreign bodies in the rectum. Usually they are inserted intentionally, however, some are inserted by mistake [2]. Sexual seduction is one of the commonest reasons for intentional insertion. Most patients with rectal foreign bodies present to the emergency room usually after efforts to remove the object at home. Many minimally invasive and invasive techniques to remove rectal foreign bodies have been described in the literature [3]. Moreover, large number of foreign body types have been described. There are very few studies in that, most of the descriptions in the available literature consist largely of case reports or case series [4].

In this report, a foreign body impacted in the rectum was removed using a foley catheter size 24. This represent a rare, but safe method of removal and few reported the use of the same technique [5].

## 2 Case report

**Fig. 1:**
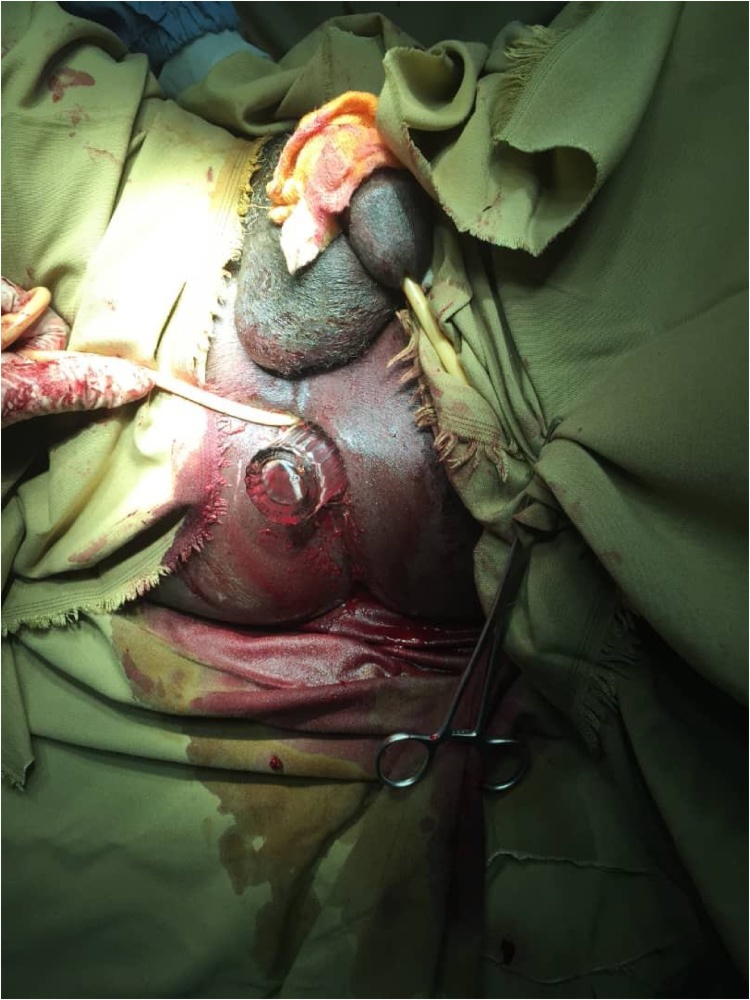
The base of the impacted foreign body (the bottle) during removal from the rectum using the Foley catheter.

**Fig. 2:**
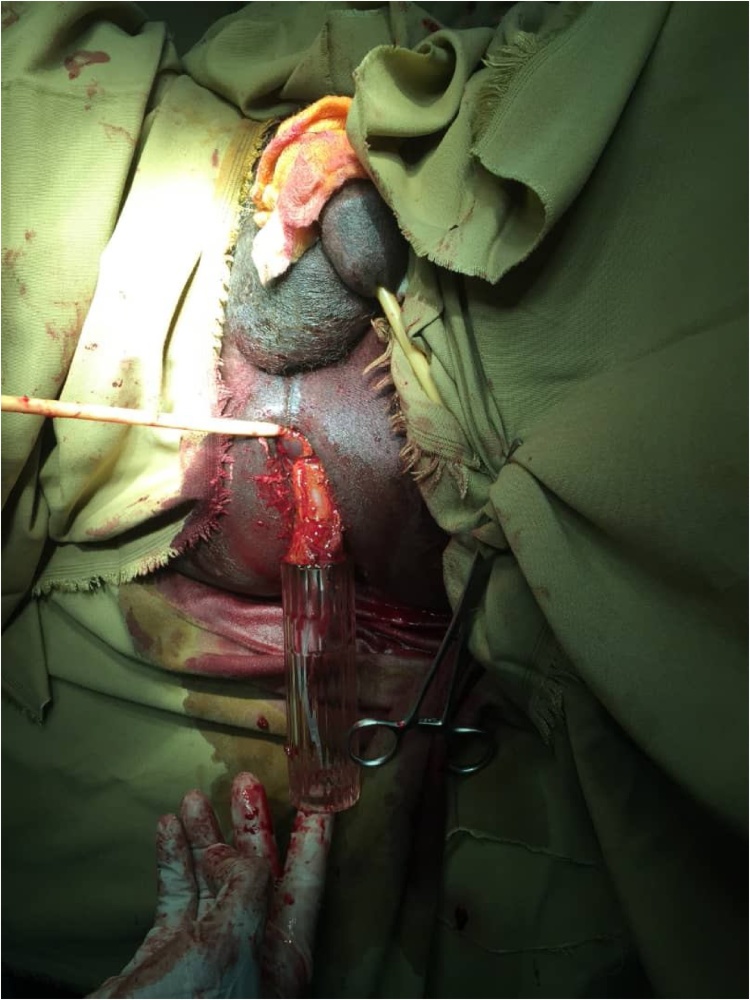
The foreign body removed from the rectum and the balloon of the catheter appears.

A 65 –year- old retired male who is not known to have any chronic organic or psychiatric disease presented to our surgical unit with anal pain and bleeding for few hour duration. The patient was looking ill and in pain. He was not pale, jaundiced or cyanosed. Abdominal examination was normal. On digital rectal examination the bottom of a foreign body was palpable and there were mild anal abrasions. The patient didn’t give a history of insertion of foreign body. He was covered with analgesia (injections of nonsteroidal anti-inflammatory drugs) and prepared for foreign body extraction under general anesthesia. Intra-operatively a trial of extraction using forceps failed, thus, a size 24 Foley catheter was used to remove the foreign body (Fig. 1). This single trial succeeded and the foreign body was a big long perfume bottle (Fig. 2). Subsequently the patient had an uneventful recovery period and he was followed up for two weeks later. He didn’t have any complications during the follow up period and serial rectal examinations were normal.

## 3 Discussion

Retained rectal foreign bodies are management dilemma. Objects are inserted mostly for sexual purposes. Other possible reasons are diagnostic or therapeutic purposes, self-treatment of anorectal disease, during criminal assault or accidents. Most objects are introduced through anus; however, sometimes, a foreign body can become swallowed and stuck in the rectum. Numerous objects, including billy clubs, various fruits and vegetables, nails, light bulbs, bottle, Impulse body spray cans, and turkey basters have been described as retained rectal foreign bodies [2].

A systematic algorithm of retrieval is essential because of the wide variation in injury caused to local tissues of the rectum. One of the most common problems faced by physicians in the management of rectal foreign bodies is the delay in presentation. Most of these patients present to the emergency room after efforts to remove the object at home [3]. Many of them also deny the history of foreign body insertion. We can notice these issues in our case.

Many surgical and non-surgical techniques have been described to remove rectal foreign bodies. Our case shows that removal of a foreign body retained in the rectum with a Foley catheter balloon inflated above the foreign body, may be an elegant and safe alternative when conventional techniques fail. It represent a less traumatic technique compared to the others. This method has been described in few reports in the literature [5].

The success of removal in this case from a single trial could be a matter of chance. More studies and case reports need to be investigated.

In the stable patient, the object can be removed in the emergency department with a regional block and/or sedation via the trans-anal approach. If this fails, then the patient should go to the operating room for a deeper anesthetic and attempt at trans-anal extraction. In difficult cases we can get the help of laparoscopy. Surgery with a laparotomy should be reserved for patients with perforation or ischemic bowel or cases of failed trans-anal attempts. After removal of the foreign body, we suggest a period of observation, a rigid or flexible endoscopy to evaluate for rectal injury. Some patients may need psychological assessment [[6], [7], [8]].

## 4 Conclusion

Foreign bodies in the rectum are not uncommon. Many methods can be used for their removal and Foley catheter extraction is one of them. It represents a safe less traumatic method of removal.

## Sources of funding

Study was not funded.

## Ethical approval

Study was approved by university of Khartoum.

## Consent

“Written informed consent was obtained from the patient for publication of this case report and accompanying images. A copy of the written consent is available for review by the editor –in-chief of this journal on request”.

## Author contribution

The first Author:

Surgical consultant and supervisor in the unit, he did the surgery and revised the case and he prepared the imaging.

The second author:

Participated in writing.

The last and corresponding author:

He wrote the case and revised it.

## Registration of research studies

Didn’t register yet.

## Guarantor

The Guarantor is the last author.

## Provenance and peer review

Not commissioned, externally peer-reviewed.

## Declaration of Competing Interest

No conflict of interest.
